# The Mixed Board Bill

**Published:** 1907-04

**Authors:** 


### The Mixed Board Bill.

A remarkable precedent was made in connection with this bill. Madame Junot said, "Some of us must be ancestors." Somebody must make a precedent, I suppose.

The bill as published in our March number was passed March 14th, and signed by the President of the Senate and the Speaker of the House and sent to the Governor. He refused to sign it, but sent it back for "amendment and correction." By some kind of *hocus pocus* it was set back in the House to "second reading," and opened for amendment. The amendments the Governor wanted were cut and dried and furnished to the champions of the bill. Then a funny thing happened.

McGregor, who did all in his power to get certain amendments put in on the first round, and to prevent the bill becoming a law, now claimed that it *was already a law* by limitation, it having been out of the hands of the Legislature eleven days (March 14th to 25th), and Jenkins held the same view in a powerful speech. Judge Neblett, our champion, and his supporters — who fought every amendment on the first round, and did all in their power to *make* the bill a *law*—now swapped ends with McGregor and Jenkins, and said it wasn't already a law, in as much as two of the eleven days were *Sundays!* They insisted on the amendments, and prevailed. The Speaker upheld the contention that Sunday, in case of a mixed board bill, is not a day, and as the bill had been in the Governor's hands only *nine days,* he ruled that it was in order to go ahead and amend it, and they did it with a rush, 76 to 28, —got them all in that the Governor had suggested.

It is now the law, and those who don't like it can lump it. It looks very much to me that the Osteos have fallen between two stools. It will require a decision of the courts or the Attorney General to tell "where they are at." Read the bill. The astute committee simply used them as a handle,— and dropped them. They got left on the last deal.

